# Loss of the Tongue

**Published:** 1875-04

**Authors:** D. R. Jennings


### LOSS OF THE TONGUE.

BY D. R. JENNINGS, D. D. S.

*Read before the Ohio State Dental Society.*

Mrs. B. came to me some time in the year of 1864 with a mouthful of very badly decayed teeth and roots. And as a natural accompaniment the whole mucus surface of the mouth congested and all the secretions very viscid and fetid. She also complained of a stiffness of the tongue or as, she said, her "tongue felt heavy and hard to handle." I extracted the roots of fifteen teeth, three incisors and two canines below, that were loosened by accumulation of tartar. Her mouth healed very rapidly and assumed a good healthy look, her breath very much improved and she expressed herself as feeling very much better than she had for a long time, only her tongue continued to grow stiff and her articulation not as good as before her teeth were extracted. In from nine to ten months from the time her teeth were extracted I made for her a full set of teeth on rubber base. And in the course of a month or so she returned to have the lower plate trimmed or something done to them as they made her throat sore, as she thought. Knowing that it was not an unusal thing for the mucus surface to be sore by irritation; from the plate being too long, I examined the mouth and could not find any soreness but found the thyroid gland somewhat enlarged over its entire surface. And the tongue stiffer than when last I saw her. I did not see her again for nearley a year when she came to the office with her daughter to have some work done. I noticed that it required quite an effort on her part then to talk intelligibly. She told me she did not think she could talk at all without the teeth, as she had lost the use of her tongue almost entirely. I examined it, but could not find any very marked difference in it from what it should have been in its normal condition. She has the sense of taste and feeling in a very good degree, but it has lost its muscular action to a great extent. On inquiry I learned that she had had several spells of spitting blood, that she thought came from the throat. I did not see her again until about two years ago. When she had lost the use of her tongue entirely, at least, so mnch so that when it was moved, it had to be done by the fingers. It still looked quite natural only perhaps some what darker in color, quite sensitive to the prick of any sharp instument.

It was with great difficulty I could understand her conversation. The next I heard was, that on the 22d day of January last, while feeding herself in her usual manner, she felt a sensation of choking. And in the effort to expel the contents of the mouth and throat she threw the tongne out with the rest. This was followed by the most severe hemorrhage she had had at any time, the appearance of the tongue was normal with the exception of the right side being some wasted. It was neither softened or indurated. The tongue had ulcerated just above the hyoid bone and seems to have done so without her being aware of such a condition existing, having no discharge of pus or anything to indicate the diseased condition until it fell out as described above, leaving the ulcerated surface in view. The ulcerated surface healed in a short time. She is feeling (now some eight months since the occurrence) as well as for years, and articulates better than when she had the paralyzed tongue. Her sense of taste is as good as ever, and she only complains of the trouble she experiences in handling her food to masticate and also to perform the act of deglutition. She relishes her food, she thinks, better than before she lost her tongue, and she has gained quite a considerable in flesh.

